# The relationship between anesthesia and melatonin: a review

**DOI:** 10.3389/fphar.2023.1255752

**Published:** 2023-09-19

**Authors:** Rui Guo, Junming Ye, Baozhen Liao, Xin Luo, Panguo Rao

**Affiliations:** ^1^ Department of Anesthesiology, First Affiliated Hospital of Gannan Medical College, Ganzhou, Jiangxi, China; ^2^ Suzhou Medical College of Soochow University, Suzhou, China; ^3^ Gannan Medical University, Ganzhou, Jiangxi, China

**Keywords:** melatonin, anesthesia, perioperative administration, perioperative sleep disturbances, perioperative anti-anxiety

## Abstract

**Introduction:** This comprehensive review delves into the intricate and multifaceted relationship between anesthesia and melatonin, aiming to provide essential insights for perioperative clinical anesthesiologists and stimulate interest in related research. Anesthesia and surgery have the potential to disrupt melatonin secretion, leading to sleep disorders, postoperative neurocognitive dysfunction and other symptoms. In comparison to previous reviews, this review provides a comprehensive summary of the various aspects linking melatonin and anesthesia, going beyond isolated perspectives. It explores the potential benefits of administering melatonin during the perioperative period, including alleviating anxiety, reducing pain, enhancing perioperative sleep quality, as well as demonstrating immunomodulatory and anti-tumor effects, potentially offering significant advantages for cancer surgery patients.

**Recent Findings:** Anesthesia and surgery have a significant impact on melatonin secretion, the hormone crucial for maintaining circadian rhythms. These procedures disrupt the normal secretion of melatonin, leading to various adverse effects such as sleep disturbances, pain, and postoperative neurocognitive dysfunction. However, the administration of exogenous melatonin during the perioperative period has yielded promising results. It has been observed that perioperative melatonin supplementation can effectively reduce anxiety levels, improve pain management, enhance the quality of perioperative sleep, and potentially decrease the occurrence of postoperative delirium. In recent years, studies have found that melatonin has the potential to improve immune function and exhibit anti-cancer effects, further underscoring its potential advantages for patients undergoing cancer surgery.

**Summary:** In summary, melatonin can serve as an adjuvant drug for anesthesia during the perioperative period. Its administration has demonstrated numerous positive effects, including anti-anxiety properties, sedation, analgesia, improved postoperative sleep, and the potential to reduce the incidence of postoperative delirium. Furthermore, its immune-modulating and anti-tumor effects make it particularly valuable for cancer surgery patients. However, further studies are required to determine the optimal dosage, long-term safety, and potential adverse reactions associated with melatonin administration.

## 1 Introduction

Melatonin (MT), also known as the pineal hormone, is an indole-like neuroendocrine hormone secreted by the pineal gland. It was first isolated from the pineal gland of cattle by Lerner in 1959. It has a short biological half-life and is primarily metabolized by the liver and excreted as 6-hydroxy-melatonin sulfate.

The secretion of melatonin by the human pineal gland exhibits a clear circadian rhythm. Melatonin secretion begins to increase at night, reaching its peak during the period from 2 a.m. to 4 a.m., while its daytime secretion remains low, almost in a resting state. This rhythmic pattern of peak and trough secretion forms the biological basis of the sleep-wake cycle (Vasey et al., 2021). Melatonin secretion levels are closely associated with age, with a gradual decline in secretion as age increases. Newborn infants have minimal melatonin secretion, which does not increase until 3 months of age, when a more pronounced circadian rhythm becomes apparent. The highest level of melatonin secretion occurs between the ages of 3 and 6 years, followed by a slight decrease during adolescence. Subsequently, melatonin secretion gradually decreases with age, particularly after the age of 35, resulting in a marked decrease in secretion. On average, melatonin secretion decreases by 10%–15% every 10 years, leading to sleep disorders and various physiological dysfunctions. By the age of 70, melatonin secretion levels drop to approximately 10% of the peak, and the circadian rhythm of melatonin secretion may disappear completely in elderly individuals, contributing to sleep disorders commonly observed in this population. Decreased melatonin secretion levels and sleep disorders are important indicators of brain aging, and several studies (Chen et al., 2021) have demonstrated a link between melatonin secretion levels and the onset of neurodegenerative diseases such as Alzheimer’s disease and Parkinson’s disease.

Melatonin’s primary physiological function is to regulate the human biological rhythm, with the circadian rhythm being the most crucial biological rhythm. Additionally, melatonin exhibits other physiological functions such as sedation and hypnosis, anti-anxiety and analgesic effects, antioxidative properties, regulation of reproductive activities, immune modulation, and effects on neuroendocrine function and aging. Currently, exogenous melatonin is predominantly used in the treatment of sleep disorders and as an adjuvant therapy in anti-aging and anti-tumor treatments. In the field of anesthesia, it is primarily employed as an adjuvant therapy for preoperative anti-anxiety and postoperative sleep improvement.

Melatonin cell membrane receptors in humans and other mammals are classified into three subtypes: MT1, MT2, and MT3. The majority of melatonin’s biological functions are mediated by the G-protein-coupled MT1 and MT2 receptors, which exhibit high amino acid homology and complementarily regulate the circadian rhythm and immune system in the central nervous system (Gobbi and Comai, 2019). The MT3 receptor belongs to the quinone reductase family and possesses potent antioxidant capacity, with its specific antagonist being prazosin. Furthermore, extracapsular membrane receptors and intracellular receptors of melatonin, belonging to the retinoid-like orphan receptor (RoR) family, have also been identified (Emet et al., 2016)^.^

## 2 Effects of anesthesia and surgery on melatonin secretion *in Vivo*

Perioperative trauma, anesthesia, stress, pain, and inflammation can lead to disruptions in the sleep-wake regulation system and circadian rhythm, resulting in circadian rhythm disturbances and decreased melatonin secretion. General anesthesia, spinal anesthesia, and commonly used clinical anesthesia drugs such as benzodiazepines, propofol, inhaled anesthesia drugs, and opioids have been found to affect melatonin secretion. Surgical trauma-induced stress and inflammation cause an increase in cortisol concentration in the body, which suppresses the activity of N-acetyltransferase, leading to a reduction in melatonin synthesis. The intensity of the stress response correlates with cortisol levels, resulting in greater suppression of melatonin synthesis and lower melatonin levels (Faassen et al., 2017).

Research studies have demonstrated that knee surgery in adults, whether under general anesthesia or spinal anesthesia, delays the onset of nighttime melatonin secretion and significantly disrupts the normal circadian rhythm of melatonin. A prospective clinical study comparing general anesthesia and lumbar anesthesia in elderly patients undergoing hip fracture surgery found that the circadian rhythm disturbance was more pronounced in the general anesthesia group compared to the lumbar anesthesia group. On the first day after surgery, the general anesthesia group exhibited significantly lower peak concentration, median value, and amplitude of melatonin secretion compared to the lumbar anesthesia group, possibly due to the lower stress response associated with spinal anesthesia. Furthermore, compared to spinal anesthesia, general anesthesia and the administered drugs significantly interfered with the circadian rhythm and melatonin secretion (Kärkelä et al., 2002; Song et al., 2021).

## 3 Melatonin for anti-anxiety and improvement of sleep disorders during the perioperative period

### 3.1 Perioperative anti-anxiety

Numerous studies have demonstrated the efficacy of melatonin in reducing perioperative anxiety. A prospective clinical study (Khare et al., 2018) found that administering 6 mg of melatonin orally to adult patients 120 min before the induction of general anesthesia provides superior anti-anxiety effects compared to administering 0.25 mg of alprazolam to adult patients. Melatonin administration resulted in less sedation and better preservation of cognitive and psychomotor function. In pediatric patients, preoperative anxiety and stress tend to be more significant, and oral administration of melatonin is considered more suitable. In a randomized, prospective, double-blind study (Kurdi and Muthukalai, 2016) involving children, four groups were formed. Each group received oral administration of melatonin at doses of 0.75 mg/kg or 0.5 mg/kg, midazolam at a dose of 0.5 mg/kg, or a placebo 45–60 min before the induction of general anesthesia. The study findings demonstrated that the group receiving melatonin at a dose of 0.75 mg/kg exhibited the lowest preoperative anxiety scores and the highest compliance during the induction of anesthesia. Although midazolam also reduced anxiety in children, it led to decreased success rates of separating children from their parents before anesthesia and negatively impacted cognitive function. The results highlight the advantages of using melatonin as preoperative medication for children, as it effectively alleviates anxiety without affecting mental and cognitive function, and enhances compliance prior to anesthesia.

A study Sane et al. (2023) involving 120 cases of cataract surgery under local anesthesia demonstrated that preoperative oral administration of 3 mg of melatonin 60 min before the surgery effectively reduced patients’ anxiety scores, pain scores, and intraocular pressure. Preoperative administration of melatonin not only alleviates perioperative anxiety but also reduces the dosage of intraoperative anesthetic propofol, improves postoperative pain management, and does not prolong postoperative recovery time. When compared to midazolam, melatonin has minimal impact on rapid eye movement sleep (REM) and psychomotor behavior, thus favoring better postoperative recovery. Animal studies (Thomson et al., 2021) on mice lacking the MT2 receptor of melatonin have demonstrated attention-deficit and anxiety-like behavior, suggesting the involvement of the MT2 receptor in cognition and anxiety regulation. Another study (Sundberg et al., 2020) investigating outpatient patients with emotional disorders measured daytime melatonin levels and inflammatory markers in saliva, confirming a significant correlation between daytime melatonin levels and anxiety disorders.

### 3.2 Association of melatonin with perioperative sleep disorders

Traumatic stress from anesthesia and surgery may affect the main neurotransmitter system related to circadian rhythm control, such as gamma-aminobutyric acid/N-methyl-D-aspartic acid (GABA/NMDA), inhibit the expression of the core clock gene per2, and interfere with endogenous melatonin secretion. It is possible that anesthesia can mimic circadian clock adaptation to day-night changes. This may lead to postoperative sleep disturbances by disrupting the circadian rhythm and causing changes in the molecular clock (Poulsen et al., 2018). Melatonin induces sleep, shortens sleep latency, prolongs sleep duration, and improves sleep quality. Melatonin binds to the widely distributed high-affinity MT1 and MT2 receptors in the suprachiasmatic nucleus (SCN) of the hypothalamus to regulate the timing of sleep. MT1 inhibits neuronal activity and induces sleep, while MT2 mainly induces a shift in sleep phase and regulates the circadian rhythm. Exogenous melatonin has been widely used in the field of sleep in the clinic. Its indications include a series of sleep problems caused by sleep disorders, old age, jet lag, night shift work, *etc.*

In a clinical study involving 100 patients who underwent laparoscopic cholecystectomy, it was found that when 6 mg of melatonin tablets or a placebo were taken 45 min before bedtime for 3 days post-surgery, the melatonin group showed an increase in total sleep time and a reduction in sleep latency on the first and second days after surgery. This indicates that melatonin can effectively improve the sleep quality of postoperative patients (Vij et al., 2018). Taking 6 mg of melatonin orally 60 min before bedtime during the perioperative period of breast cancer surgery significantly improved sleep efficiency and reduced wakefulness after sleep for the whole 2 weeks after surgery (Madsen et al., 2016). During patients after craniotomy, studies (Arık et al., 2020) have shown that noise and light isolation measures such as eye masks and earplugs can increase the secretion of melatonin after surgery, thereby improving sleep.

The pineal gland of elderly individuals atrophies, its function degrades, melatonin secretion levels are reduced, and the circadian rhythm of secretion changes or even disappears, so postoperative sleep disorders are more serious in this population. In an animal study (Jia et al., 2021) of elderly mice, which were divided into a melatonin group and a control group, a sevoflurane inhalation anesthesia group and a sevoflurane anesthesia-exploratory laparotomy group were set up at the same time. The research results showed that sevoflurane anesthesia-laparotomy had a greater effect on postoperative sleep than sevoflurane anesthesia alone. Compared with sevoflurane exposure alone, laparotomy under anesthesia resulted in greater changes in sleep latency, sleep duration, sleep power, and structure, suggesting that surgery had a more profound effect on postoperative sleep. While melatonin preconditioning improved postoperative changes in sleep and wake times and reversed the circadian rhythm fluctuations caused by sevoflurane anesthesia. Melatonin can reduce the number and duration of wake attacks after sevoflurane anesthesia, suggesting that melatonin can effectively reduce sleep disorders after sevoflurane anesthesia in elderly mice. Propofol, an intravenous anesthetic commonly used in the clinic, can activate the CAMK-CREB signaling pathway, thereby inhibiting the expression of the circadian rhythm factors PER and CRY in the hypothalamus and leading to sleep disorders. Melatonin preconditioning can regulate the circadian rhythm in rats to prevent sleep disorders induced by propofol (Yin et al., 2022).

## 4 Analgesic effects of melatonin

The analgesic effects of melatonin have been well-documented in various studies. During cataract surgery under local anesthesia, preadministration of 500 mg of acetaminophen or 6 mg of melatonin 60 min before the surgery has been shown to reduce surgical pain and the need for supplemental fentanyl (Haddadi et al., 2018). Similarly, it has been found that pretreatment with either 6 mg of melatonin or 600 mg of gabapentin administered 100 min before surgery effectively reduces anxiety and pain during lumbar surgery (Javaherforooshzadeh et al., 2018). In the context of caesarean section under lumbar anesthesia, a double-blind randomized controlled clinical study confirmed that preadministration of melatonin 10 mg is safe and can reduce pain severity, prolong postoperative analgesia, decrease analgesic requirements, and facilitate early mobilization (Kiabi et al., 2021). In addition, it has been demonstrated that oral administration of 6 mg of melatonin 60 minbefore surgery is effective in reducing postoperative pain, morphine consumption, the need for additional analgesics, as well as the incidence of nausea and vomiting in major abdominal surgery (Laflı Tunay et al., 2020).

Furthermore, melatonin exhibits analgesic effects in chronic pain conditions. A study revealed its significant analgesic effect in postherpetic neuralgia, possibly through the involvement of the δ opioid receptor, nitric oxide (NO), and MT2 receptors (Deng et al., 2015). Another study utilizing a rat model of neuropathic pain demonstrated that the MT2 receptor agonist IIK-7 can alleviate mechanical abnormal pain and inhibit glial cell activation, as well as various proteins related to inflammation and apoptosis (Kutha et al., 2019).

Overall, existing studies have established the analgesic effects of melatonin in perioperative acute pain, as well as inflammatory and chronic neuropathic pain. However, the precise mechanisms underlying these effects remain unclear. It is postulated that melatonin receptors (MT1 and MT2) located in the dorsal region of the spinal cord and different brain regions involved in pain regulation may play a role (Srinivasan et al., 2012).

## 5 Melatonin and neurocognitive impairment after surgery

Postoperative neurocognitive impairment encompasses two main conditions: postoperative delirium (POD) and postoperative cognitive dysfunction (POCD). POD refers to a transient dysfunction of the central nervous system that occurs shortly after surgery (within 7 days, typically within 1–3 days), while POCD refers to a more general decline in cognitive function that manifests 2 weeks or more after surgery (Olotu, 2020).

Various factors, such as perioperative trauma, anesthesia, stress, pain, and inflammation, can affect the sleep-wake regulatory system and disrupt the circadian rhythm, leading to a reduction in melatonin secretion. General anesthesia, spinal anesthesia, and many anesthesia drugs can disturb the circadian rhythm. Consequently, the postoperative sleep-wake cycle and melatonin secretion in patients become desynchronized, contributing to the development of postoperative neurocognitive disorders (Brainard et al., 2015).

Elderly individuals already exhibit reduced pineal function and melatonin secretion, as well as preexisting sleep-wake cycle disturbances. Surgery and anesthesia further impact melatonin secretion, exacerbating the desynchronization of the circadian rhythm and endogenous melatonin. As a result, elderly patients are more susceptible to experiencing POD or POCD (Chakraborti et al., 2015).

Animal experimental studies (Song et al., 2018) have demonstrated that melatonin can prevent cognitive disorders induced by isoflurane by promoting clock gene resynchronization and restoring the normal circadian rhythm of physiological activities and temperature. The MT2 receptor, which plays a key role in circadian rhythm regulation, is also involved in circadian rhythm disturbances observed in Alzheimer’s disease.

In pediatric patients, sevoflurane inhalation anesthesia is more likely to cause postoperative delirium and agitation compared to propofol intravenous anesthesia. Singla et al. (2021) conducted a study comparing the effects of preoperative oral administration of melatonin 0.3 mg kg^-1^, midazolam 0.3 mg kg^-1^, or placebo on POD after sevoflurane anesthesia in children. The results showed that the melatonin group had a 23.3% reduction in the incidence of POD compared to the placebo group. Furthermore, melatonin exhibited a 29.2% reduction in the incidence of POD compared to midazolam, indicating its significant potential in reducing delirium occurrence following sevoflurane anesthesia in children.

In older rats, pretreatment with melatonin has shown promise in alleviating isoflurane anesthesia-induced hippocampal beta-amyloid production and cholinergic dysfunction. Melatonin interacts with beta-amyloid protein (Aβ) production, inhibiting beta-folding and amyloid fibrinoid formation. It also enhances hippocampal choline acetyltransferase (ChAT) expression, thereby reducing postoperative cognitive impairment (Ni et al., 2013)^.^

A study (Wu et al., 2014) involving 97 elderly patients aged 65–80 years who underwent major abdominal or orthopedic surgery demonstrated that fluctuating levels of 6-sulfatoxymelatonin (6-SMT), as observed by monitoring melatonin and its metabolite urine 6-SMT, were significantly associated with an increased incidence of POCD. This suggests that fluctuating endogenous melatonin levels are linked to the occurrence of POCD.

Clinical study (Artemiou et al., 2015) indicate that administering melatonin at a dose of 5 mg every night starting from the night before cardiac surgery and continuing until the third postoperative day can significantly reduce the occurrence of postoperative delirium in cardiac surgery patients. The melatonin group exhibited a significantly lower incidence of postoperative delirium compared to the control group. Anesthesia drugs can cause nervous system damage, including oxidative stress, hippocampal injury, and disrupted synaptic formation. Melatonin’s strong antioxidant capacity allows it to scavenge free radicals and reduce oxidative stress and damage induced by isoflurane anesthesia. Additionally, melatonin reduces the expression of inflammatory factors in brain tissue and decreases postoperative neurocognitive impairment (Wei et al., 2022).

The latest systematic review (Barnes et al., 2023)and meta-analysis on melatonin’s reduction of postoperative delirium in adults included literature published from January 1990 to April 2022. The results indicate that melatonin can lower the incidence of postoperative delirium in adult surgical patients. However, there were inconsistent findings among the included studies. Therefore, further investigation is needed to establish the optimal dosing regimen for melatonin and to enhance the evaluation system for its effectiveness in medication.

## 6 Enhancement of immune function

Anesthesia and surgical trauma can weaken the body’s immune function, which is particularly detrimental for cancer patients. Melatonin can stimulate the immune system, enhance the activity of T lymphocytes, boost overall immune function. It also exhibits direct anticancer properties by inhibiting cancer cell proliferation and inducing apoptosis (Reiter et al., 2017; Moradkhani et al., 2020).

## 7 Conclusion

In recent years, melatonin and its receptors have garnered increasing attention, with research indicating its potential relevance to various health conditions such as diabetes, obesity, cardiovascular diseases, central nervous system disorders, and psychiatric conditions including autism and depression. Notably, recent studies (Vlachou et al., 2021; Hasan et al., 2022) have demonstrated the positive role of melatonin as an adjuvant in reducing the incidence and mortality of sepsis, as well as its potential as an adjunct treatment for COVID-19.

The sedative, anti-anxiety, and analgesic effects of melatonin highlight its close association with anesthesia. Currently, there is limited research on melatonin in the field of perioperative anesthesia. However, the disruption of melatonin secretion caused by perioperative trauma and anesthesia is strongly linked to postoperative sleep disorders, neurocognitive dysfunction, and other complications. The anxiolytic, sedative, and analgesic properties of melatonin make it a promising adjunct drug in perioperative anesthesia. Furthermore, being an endogenous hormone in the body, melatonin possesses a higher level of safety in clinical applications.

In addition, melatonin has demonstrated its anticancer properties and its ability to enhance immune function. Consequently, it exhibits a conspicuous advantage in its application for perioperative medication in cancer patients. Currently, there is limited research on melatonin’s utilization during the perioperative period for malignant tumor patients. It is imperative to initiate investigations into melatonin’s application in the perioperative phase for diverse cancer patients. Further, these studies should be augmented with animal experiments and *in vitro* cell experiments to delve deeper into its mechanistic actions and clinical efficacy.

In conclusion, the administration of melatonin during the perioperative period shows promising prospects. Nonetheless, it necessitates further evaluation to determine the optimal dosage, long-term safety profile, and potential adverse reactions associated with its utilization.

## 8 Strengths and limatations of this study

➢ This review offers a relatively comprehensive and up-to-date assessment of the relationship between melatonin and anesthesia.
➢ This comprehensive review provides a thorough summary of the connections between melatonin and various aspects of anesthesia, whereas previous reviews has focused on only one aspect.
➢ Proposing a fresh perspective: The administration of melatonin during the perioperative phase of malignant tumors offers more prominent benefits.
➢ The results of this review are limited by the selection of literature and the limitations posed by the heterogeneity of the included studies.
➢ Language restrictions to English may have led to exclusion of additional studies.
